# Knowledge sharing among healthcare infection preventionists: the impact of public health professionals in a rural state

**DOI:** 10.1186/1756-0500-5-387

**Published:** 2012-07-28

**Authors:** Timothy Wiemken, Philip M Polgreen, W Paul McKinney, Julio Ramirez, Emily Just, Ruth Carrico

**Affiliations:** 1 University of Louisville Division of Infectious Diseases, 501 East Broadway Suite 380, Louisville, KY, 40202, USA; 2 University of Iowa Department of Infectious Diseases, 200 Hawkins Drive, Iowa City, IA, 52242, USA; 3 Department of Health Promotion and Behavioral Sciences, University of Louisville School of Public Health and Information Sciences, 485 East Gray Street, Louisville, KY, 40202, USA; 4 University of Louisville Center for Health Hazards Preparedness, 485 East Gray Street, Louisville, KY, 40202, USA

**Keywords:** Knowledge sharing, Communication, Public health department, Healthcare-associated infections, Social network, Key player, Rural health, Information

## Abstract

**Background:**

Healthcare-associated infections are a major source of morbidity and mortality in the United States. Infection Preventionists (IPs) are healthcare workers tasked at overseeing the prevention and control of these infections, but they may have difficulties obtaining up-to-date information, primarily in rural states. The objective of this study was to evaluate the importance of public health involvement on the knowledge-sharing network of IPs in a rural state.

**Findings:**

A total of 95 attendees completed our survey. The addition of public health professionals increased the density of the network, reduced the number of separate components of the network, and reduced the number of key players needed to contact nearly all of the other network members. All network metrics were higher for public health professionals than for IPs without public health involvement.

**Conclusions:**

The addition of public health professionals involved in healthcare infection prevention activities augmented the knowledge sharing potential of the IPs in Iowa. Rural states without public health involvement in healthcare-associated infection (HAI) prevention efforts should consider the potential benefits of adding these personnel to the public health workforce to help facilitate communication of HAI-related information.

## Introduction

Healthcare-associated infections (HAIs) are a major source of morbidity and mortality, causing an estimated two million infections and 100,000 deaths each year [1]. From a public health perspective, the number of HAIs exceeds the number of cases of any notifiable disease, and the deaths associated with HAIs are greater than the number of deaths attributable to several of the top ten leading causes of death [1]. The importance of HAIs to public health practice was highlighted by the addition of a Healthy People 2020 goal to “prevent, reduce, and ultimately eliminate healthcare-associated infections (HAIs) ” [2]. The steady increase in the number of states with legislative mandates for the reporting of HAIs has increased the need for communication between healthcare, public health, and government in terms of identifying HAIs, reporting HAIs, and improving patient outcomes.

Existing relationships between healthcare facility infection preventionists (IPs) and their public health colleagues varies greatly from state to state. States such as New York, California, and Pennsylvania have sophisticated approaches to collaboration that have resulted in advances in prevention efforts [3,4]. In rural states, where a substantial number of hospitals are small critical access hospitals, public health departments and IPs are confronted with unique challenges. For example, with the exception of a few studies, [5-9] basic epidemiologic data on HAIs in rural states are missing from the literature. These lack of data prevent many targeted interventions from ever being realized. Furthermore, rural states may have limited resources compared to more metropolitan states, and therefore may be forced to leverage existing relationships to implement interventions that rely heavily upon their current capabilities. The Commonwealth of Kentucky provides an example of such a rural state.

Kentucky has a population of approximately 4 million [10] and is 56% rural [11]. By comparison, the US is considered to be 19% rural [11]. A recent survey of Kentucky’s IPs revealed that 25% have less than 5 years of experience in that role, [12] they lack formal mentoring programs, and their job responsibilities often exceed their education and experiential preparation [13]. In order to address this training and knowledge gap, it is critical to understand how knowledge is shared among these professionals. An analysis of the existing knowledge-sharing network in Kentucky was conducted in 2010, and found that there were distinct gaps in the ability of the IPs to gain and share knowledge with respect to infection prevention and control [12]. One of the recommendations from this study was to define ways to increase knowledge sharing between IPs.

The state of Iowa is similar to Kentucky in that it is predominantly rural (70%) and has a similar configuration of hospital sizes [14]. Iowa differs from Kentucky in that they have long-standing, state wide healthcare-public health partnerships. These partnerships may provide a network structure more conducive to knowledge sharing, resulting in decreased efforts necessary in conducting and coordinating infection prevention activities.

The purpose of this study was to define the role of public health professionals on the knowledge-sharing network of IPs in a rural state.

## Methods

### Study design and population

A social network analysis was conducted to evaluate the impact of public health professionals on the knowledge-sharing network of IPs in Iowa. A whole network approach was used [15]. A paper-based survey was provided to all attendees at a statewide infection control meeting in Iowa in May 2011. Instructions were given orally and were included on the survey instrument. Completed surveys were collected approximately 30 minutes after initiation. The survey has been previously utilized in another communication network protocol [16] and was pilot tested prior to the previous study and the current study.

### Study definitions

The analysis methodologies used in this study require definition for an adequate understanding of the study results. Brief definitions for the results reported below can be found in Table 1. In-depth discussions of the concepts can be found elsewhere [17,18].

**Table 1:** Study definitions

**Term**	**Definition**
Alter	A member of the network with whom another member of the network shares knowledge, an IPs communication contacts [here, used only in the definition of eigenvector centrality].
Authority	A member of the network that receives communication from many hubs. This person receives information from potentially important people in the network.
Betweenness Centrality	How often a member of the network falls between the shortest knowledge-sharing path of two other members of the network. For example, if two IPs have a mutual contact but cannot, themselves, communicate with one another, their mutual contact serves as a broker of communication, a network node with high *betweenness*, as he or she falls within the shortest communication path between the other two network members.
Bridge	A node connected to a diverse set of other nodes
Centralization	How much the network is centered around a few central members.
Clique	A subgroup of members of the network in which each person is connected to every other member of the subgroup.
Component	A sub-group that was not connected to any sub-group in the network.
Constraint	A measure of bridging: low constraint indicates connection to others who are not themselves connected.
Eigenvector Centrality	The relative number of knowledge-sharing episodes of a particular member of the networks alters. For example, if an IP#1 has one alter (IP#2) who themselves has one alter (IP#3), the maximum amount of knowledge that can be gained by IP#1 is, at most, the sum of the knowledge of IP#2 and IP#3 (low eigenvector centrality for IP#1). However, if IP#2 has 80 contacts, the knowledge potential through the IP#1 to IP#2 communication path is much greater since IP#2 has a lot of different communication paths with which to obtain information (high eigenvector centrality).
Fragmentation Key Player	A member of the network capable of holding the network together in as few components as possible while taking into account the size of each component as well as the other fragmentation key players. This is an IP that, if removed can break up the network into many pieces, cutting off certain members from obtaining information from the rest of the network.
Hub	A member of the network that provide information many authorities. These members provide information to potentially important members of the network.
In-degree Centrality	The number of times a member of the network was asked for knowledge.
Isolate	A member of the network with no connections to any other member of the network.
Knowledge Sharing	Having exchanged infection prevention-related information formally or informally via any method with another member of the network in Iowa over the past 6 months.
Node	A survey respondent.
Out-degree Centrality	The number of times a member of the network asked another member of the network for knowledge.
Reach Key Player	A member of the network that had the capability of sharing knowledge with the largest proportion of other nodes in the network while taking into account the other reach key players and the potential for redundant connections. This is an IP that has a lot of contacts that other members of the network do not share, making them a unique individual with respect to the number of other members of the network with which they can communicate.

### Statistical analysis

Network cohesion was measured by *densityclique*, and *component* analyses. *Node* centrality was measured using *in-degreeout-degreebetweenness*, and *eigenvector* centrality measures. *Centralization* indices were used to demonstrate the level to which the network focused on one particular central node. *Bridges* were identified using the *constraint* statistic [19]. *Hub* and *authority* scores were calculated using generalizations of eigenvector centrality [20]. Key players in the network were identified using the key player algorithm developed by Borgotti and colleagues [21]. Both *reach* and *fragmentation key players* were identified using sequential addition of key players. The final number of key players in each analysis was identified when the reach index or fragmentation index did not increase more than 10% with the addition of the extra key player [12]. The multiple group sizes algorithm was used to compute the total number of key players necessary to reach 100% of the other members of the main component of the network. UCINET and NETDRAW were used for all social network analyses (Analytic Technologies, Lexington, KY). KeyPlayer v1 was used for identification of key players (Analytic Technologies, Lexington, KY). SAS v9.2 (SAS Inc. Cary, NC) was used for other descriptive analyses.

To evaluate the impact of the public health department coordination in Iowa, all analyses were conducted twice: once with only the IPs and once with the IPs and the health department personnel.

### Human subjects protection

Institutional review board approval was granted by the University of Louisville Human Subjects Protection Office (Protocol #08.0399) prior to any data collection. Written informed consent was waived for all data collection activities, and no data were collected from children.

## Findings

**Figure 1:**
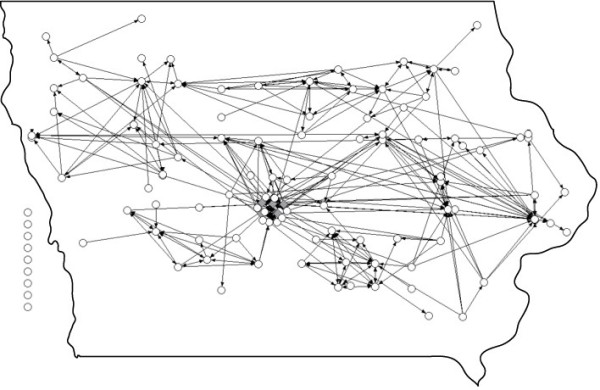
** The knowledge sharing social-network of healthcare infection preventionists and public health department personnel in Iowa [Public health personnel are indicated with darker, square nodes in the center of the figure – node locations are geocoded based on the primary site of employment and are approximate].** Note: Geocoding limits the ability to distinguish network components.

A total of 95 attendees completed the survey instrument, of which 4 were public health professionals (4%). This represents 66% of the public health workforce that is involved in infection prevention activities. Figure 1 depicts the knowledge-sharing social network of survey respondents, geocoded to their primary site of employment.

Demographic characteristics of respondents identified to be IPs can be found in Table 2. The plurality of survey respondents were in the 46-55-year age group (39%), were female (95%), and had a 4-year college degree (41%). A total of 23% were certified in infection control through the Certification Board of Infection Control and Epidemiology (CBIC). One third of respondents indicated that they had practiced in their current position for over 10 years, and almost half indicated having practiced infection prevention for over 10 years. Over two thirds of respondents specified having less than 100% of their daily efforts devoted to the practice of infection prevention.

**Table 2:** Demographic characteristics of 91 responding infection preventionists in Iowa

**Variable**	**n (%)**
Age Group	
26-45	29 (31.9)
46-55	35 (38.5)
56-65	25 (27.5)
>65	2 (2.2)
Female Gender	86 (94.5)
Education	
2-3 Year College (Including RN)	35 (38.5)
4 Year College	37 (40.7)
Master Degree	16 (17.6)
Professional Degree (ARNP, PA, etc.)	2 (2.2)
CIC Certification	22 (23.3)
APIC Member	69 (76.7)
Regular APIC Meeting Attendance – Local Chapter	51 (56.7)
Regular APIC Meeting Attendance – Statewide Meeting	61 (67.0)
Time in Current Position	
Less than 1 Year	11 (12.1)
1-2 years	10 (11.0)
3-5 years	22 (24.2)
6-10 Years	19 (20.9)
More than 10 Years	29 (31.9)
Daily Efforts in Infection Prevention	
<20%	20 (22.0)
20%-50%	26 (28.6)
More than 50% but less than 100%	17 (18.7)
100%	28 (30.8)
Length of Career in Infection Prevention	
Less than 1 Year	10 (11.1)
1-2 years	7 (7.8)
3-5 years	15 (16.7)
6-10 Years	16 (17.8)
More than 10 Years	42 (46.7)
Affiliation with an Academic Center	15 (16.5)
Use of the National Healthcare Safety Network	60 (65.9)

Table 3 reports the centrality and centralization statistics for the knowledge-sharing network for IPs and for the public health professionals. All network statistics were higher for public health professionals than for IPs without public health involvement. Furthermore, the in-degree and betweenness centralization indices were higher for public health professionals (in-degree 13%, betweenness 10%) than for IPs (in-degree 9%, betweenness 6%).

**Table 3:** Centrality and centralization statistics of the knowledge-sharing social network of hospital-based infection preventionists and public health pofessionals in Iowa

**Variable**	**Median (Range)** **IPs**	**Median (Range)** **Public Health**	**Centralization** **IPs**	**Centralization** **Public Health**
In-degree	1 (0–13)	4.5 (3 – 19)	9%	13%
Out-degree	4 (0–5)	4.5 (2–5)	2%	2%
Betweenness	1.3 (0 – 950.5)	923.0 (509.2-1406.2)	6%	10%
Eigenvector	0.04 (0–0.32)	0.02 (0.13 – 0.32)	44%	40%
Hub	0.04 (0 – 0.28)	0.07 (0.05-0.14)	-	-
Authority	0.01 (0 – 0.47)	0.13 (0.08 – 0.57)	-	-

Table 4 describes the cohesion and key player statistics for the network. The network with the public health professionals increased the network density from 1.7% to 1.9%, and reduced the number of *isolates* and components. In the network without the public health professionals, 11 reach key players were able to contact just over 70% of the network. In the network with the addition of public health professionals, two less reach key players were needed (n = 9) to reach a similar proportion of other members of the network. Furthermore, two fewer reach key players (n = 23 versus n = 21) were needed to reach 100% of the other members of the main component of the network. Four meaningful fragmentation key players were found in the network without the public health professionals and when removed, produced a 54% fragmentation index. Three meaningful fragmentation key players were found with the addition of the public health professionals, and produced a 34% fragmentation index upon removal. Finally, the lowest non-zero constraint score was held by a public health professional (0.083).

**Table 4:** Cohesion and key player statistics for the knowledge-sharing social network of hospital-based infection preventionists and public health professionals in Iowa

**Variable**	**Value without** **Public Health**	**Value with** **Public Health**
Cohesion		
Density	1.7% (274 ties)	1.9% (336 ties)
Isolates	n = 11	n = 9
Components	n = 3	n = 2
Cliques	n = 4	n = 4
*Key Players*		
Reach Key Players	n = 4 (72% Reachable)	n = 3 (74% Reachable)
For 100% Reach of Main Component	n = 23	n = 21
Fragmentation Key Players	n = 4 (54% Fragmentation)	n = 3 (34% Fragmentation)

## Discussion

Our results demonstrate that complementing the IP knowledge network with public health professionals may increase the ability of IPs to share knowledge with each other. Since many IPs in Iowa communicate with the public health department regarding HAIs those public health professions play a unique role within the structure of the Iowa network. Our findings that public health professionals in Iowa have higher network statistics suggest that these members have important structural roles within the infection prevention knowledge network. The higher in-degree statistics for public health professionals suggest that they are contacted by many IPs, while higher out-degree statistics suggest they provide information to many members of the network. The larger betweenness statistics suggest public health professionals can connect otherwise disconnected members of the network, and higher eigenvector statistics suggest that these members contact IPs who themselves contact a large number of other IPs. These findings provide evidence that information can quickly diffuse through this network when provided to public health professionals.

One important finding was that public health members are able to connect otherwise isolated IPs. Although the density of the network did not increase a great deal (0.2%) with the addition of public health professionals, they added 62 communication ties to the network, a very large number for only 4 additional members. These additional ties will allow network members to maintain communication if some IPs leave their positions. Key player metrics further demonstrated the importance of these members within the knowledge-sharing network. With the addition of the public health members, communication between nearly every member of the network was possible after initiating communication through a smaller set of key members. In the event of a pandemic or outbreak, it is critical to quickly convey new information to nearly every member of the network, and focusing communication efforts on these reach key players may provide an ideal mechanism for improving the flow of information. Finally, we found that the public health members reduced the network fragmentation upon removal of fragmentation key players. Because of the presence of these public health professionals and their unique placement within the structure of the network, the network will be less affected when other members leave the network (e.g. retirement or changing jobs).

Another important concept in communication networks is “bridging”. Members who represent bridges are capable of connecting members of a network that do not readily connect with each other. These members are considered to be personnel with critical influence for improving team performance in a network [19,22,23]. Here, we identified that the network member with the lowest constraint score (indicative of a good bridge) was a public health professional. This bridge allows for dissemination of information from diverse areas of the network. As various areas of the network may hold members with different areas of expertise, bridges allow for this expertise to flow to and from these areas. For example, if some IPs in a network are strong in prevention of ventilator-associated pneumonia, and another is proficient with use of the National Healthcare Safety Network (NHSN), the network bridge is capable of connecting these IPs. Bridges are particularly important in a rural state where most of the hospitals are critical access hospitals. In these facilities, IPs have multiple demands and limited resources, making it difficult to gain expertise in multiple areas. Because of these factors, it is especially important to identify bridging network members capable of gathering and disseminating a wide variety of HAI-related information.

Another possible method of increasing knowledge sharing is periodic statewide meetings of infection preventionists [12]. However, despite the presence of a long-standing annual statewide IP meeting in Iowa, there were many similarities between the Iowa and the Kentucky IP knowledge-sharing networks. Both of these knowledge-sharing networks had similar numbers of components, similar densities and similar skewed node centrality scores. Also, these networks shared similar numbers of reach key players that could reach approximately the same proportion of other IPs in the network [12]. These findings were surprising, as we originally thought that the longstanding statewide meeting in Iowa would have led to a much different more connected network in Iowa compared to Kentucky. Iowa does have regular APIC chapter meetings, which are similar to the structure within Kentucky. These local chapter meetings may also be a mechanism for communication in this group of professionals outside of the statewide meeting. This suggests that local APIC chapter meetings function similarly in both Iowa and Kentucky with regard to knowledge sharing. However, it appears that the only major differences in the networks are the addition of the public health professionals.

Our study has several limitations, including the possibility of missing data. As not every member of the knowledge-sharing network attended the statewide meeting, it is possible that important members of the network were not included in this analysis, thereby biasing the results. A basic assumption in knowledge sharing is that the information that is shared is correct, which may not be the case. The knowledge network may be responsible for the sharing of misinformation that may, in fact, be detrimental to HAI elimination efforts. Although generalizability to other states may be limitation, it is important to emphasize that public health professionals may play important functional roles for bolstering communication in metropolitan states as well as rural states. The ability of these professionals to focus infection prevention activities, as well as their key placement in public health departments suggests that their roles in supporting communication may apply to all types of facilities in all states.

Despite these limitations, our results demonstrate that public health officials play an important role in the communication network among IPs in Iowa. Iowa and Kentucky are similar in terms of the percentage of population living in rural regions, but in Iowa the state health department is more involved with coordinating HAI prevention. Future research efforts should be devoted to discovering what kinds of information travel across these communication networks, and understanding how the metrics we present affect real-world knowledge sharing.

## Conclusions

In conclusion, our results suggest that the addition of public health professionals involved in the coordination of healthcare infection prevention activities may augment knowledge sharing among IPs in rural states. Given the importance of HAIs, rural states without public health involvement in infection prevention should consider the potential benefits of adding these personnel to the public health workforce.

## Competing interests

The authors do not have any conflicts to report.

## Authors’ contributions

Protocol development: TW, PP JR, RC. Data Collection: TW, EJ, PP. Data Analysis: TW, EJ, PP, RC. Manuscript Development/Critical Revision: TW, PP, WPM, JR, EJ, RC. All authors read and approved the final manuscript.
